# Physicochemical Properties of Biochars Produced from Biosolids in Victoria, Australia

**DOI:** 10.3390/ijerph15071459

**Published:** 2018-07-10

**Authors:** Yumeng Yang, Barry Meehan, Kalpit Shah, Aravind Surapaneni, Jeff Hughes, Leon Fouché, Jorge Paz-Ferreiro

**Affiliations:** 1 School of Science, RMIT University, GPO Box 2476, Melbourne 3001, VIC, Australia; s3481571@student.rmit.edu.au (Y.Y.); barry.meehan@rmit.edu.au (B.M.); jeff.hughes@rmit.edu.au (J.H.); 2 School of Engineering, RMIT University, GPO Box 2476, Melbourne 3001, VIC, Australia; kalpit.shah@rmit.edu.au; 3 South East Water Corporation, Waters Edge, 101 Wells Street, Frankston 3199, Victoria, Australia; Aravind.Surapaneni@sew.com.au (A.S.); leon.fouche@sew.com.au (L.F.)

**Keywords:** biochar, pyrolysis, biosolids, heavy metals, phosphorus

## Abstract

Some of the barriers associated with the land application of biosolids generated in wastewater treatment plants can be eliminated simply by converting the biosolids into biochar using a thermal conversion process called “pyrolysis”. In the current work, eight biosolids from four different wastewater treatment plants in southeast Melbourne, Victoria, Australia were collected and pyrolysed to produce biochars at two different temperatures (500 and 700 °C). In addition, characterisation studies were carried out on the biochars to obtain their physicochemical properties, which were subsequently compared with the properties of the parent biosolids. The major findings of the work demonstrated that biochars exhibited large decreases in DTPA-extractable metals such as Cd, Cu, and Zn, and also led to favorable changes in several chemical and physical characteristics (i.e., pH, Olsen P, electrical conductivity, and surface area) for agricultural land application compared to their original form (i.e., biosolids). Overall, the study suggests that there is great potential for converting biosolids to biochar using pyrolysis. This may not only improve the properties of biosolids for land application, but also has potential to reduce the risk to receiving environments and, furthermore, eliminate many of the costly elements associated with biosolids stockpiling and management.

## 1. Introduction

Biosolids, also referred to as treated or stabilised sewage sludges, are generated from the biological treatment of wastewaters. The amount of biosolids produced is increasing every year as a consequence of the expansion in the number of households connected to wastewater treatment plants (WwTPs). For example, production in Australia has increased from 300,000 tonnes per year of dry solids in 2010 to 327,000 tonnes per year of dry solids in 2017 [1]. Victoria, New South Wales, and Queensland are the major contributors and yield 31%, 25%, and 21% of the total amount, respectively [1]. Subsequently, it is becoming an imperative to find innovative approaches to handle these materials more effectively.

Biosolids constitute potential resources for agricultural production because they contain organic matter, nutrients, and trace elements [2,3]. However, costly and limited storage capacity for biosolids stockpiling, and perceived concerns around soil contamination, odours, and pathogens have added significant challenges to the management of biosolids for WwTPs. For example, according to EPA Victoria (Australia) guidelines for biosolids management, newly-generated biosolids must be stockpiled (stored) on site for at least 3 years, as a treatment process, in order to meet the biosolids classification of treatment grade of T1, which is the highest treatment grade [4]. Achieving the T1 treatment grade is necessary to market the biosolids to agricultural customers as the outlets for lower grade products (T2 and T3 grades) are very limited. In addition, the land application of biosolids can result in (i) the contamination of land with heavy metals (Cu, Zn, and Cd); (ii) excessive nutrient buildup in soils, particularly P [3]; and (iii) the potential transfer of emerging contaminants such as plastics, micro-beads, and chemicals to the terrestrial environment [5].

Pyrolysis, a thermal conversion process, can produce a carbon-rich solid material called “biochar” from biomass or related organic wastes. Pyrolysis is a way to reduce the volumes of waste streams while creating a valuable product for agricultural purposes. Pyrolysis offers significant benefits to the sustainable management of biosolids, compared to their direct land application, landfilling, or incineration. Most of the problems, when biosolids are applied directly to the soil, including the presence of pathogens and odours (especially with lower treatment grades), can be eliminated or minimised by pyrolysis. The operational parameters of the pyrolysis process and the intrinsic properties of organic matter in biomass/organic waste play an important role in controlling the quantity and quality of the final biochar product [6,7]. The highest temperature reached during pyrolysis and the duration of the process can have a direct impact on the yield and properties of biochar. A higher pyrolysis temperature and a longer duration will decrease the biochar yield, but will also increase the fixed carbon, surface area, and pore size [7,8].

Much of the initial work on the pyrolysis of biomass/organic waste has focused on the recalcitrant pools of carbon imbued in the resulting product, biochar. Thus, biochar has the potential for long-term C (i.e., carbon) storage in soils, diverting biomass C from a rapid to a slow C-cycling pool [9]. However, recent research has identified multiple benefits from soil amendment with biochar, which usually result in an increase in crop yields [10]. Most biochars, including those prepared from biosolids, exhibit neutral to alkaline properties depending on their pyrolysis temperatures [11,12], and this can result in a shift towards more neutral pH in acidic soils. Biochar derived from biosolids retains some of the necessary nutrients for plant growth as they are relatively non-volatile [13]. Previous work has demonstrated the benefits of using biochar prepared from biosolids as a substitute or in conjunction with conventional fertilisers for agronomic purposes [14]. For example, increases of yields up to 64% have been reported for cherry tomato after the application of this type of product [13].

Biochars derived from biosolids have also shown significant reduction in the plant available metal contents, including Zn, Pb, Cu, and Cd [12,13]. This effect has been reported in biochars prepared from a multitude of feedstocks (sewage sludge, chicken manure, rice straw, wheat straw, etc.) and can be attributed to several mechanisms, including surface binding to the functional groups of the biochar, ion exchange reactions between the biochar and the soil solution, the electrostatic attraction of heavy metals, and the precipitation of the metals [15].

It is expected that for land applications, biochar produced from biosolids using pyrolysis will be more stabilised compared to feed material biosolids. The pyrolysis temperature could also have a major effect on the stability and physicochemical properties of biochar [14,16]. However, pyrolysis is an endothermic process and a higher pyrolysis temperature would require higher energy for the process.

This study incorporates a range of biosolids (collected from different unit operations and locations and with varied stockpile duration) in the pyrolysis process for the first time. The aim is to assess the physicochemical properties of the biochar produced from various biosolids. This represents a scenario in which pyrolysis is used to reduce biosolids inventory at a particular WwTP where different types of biosolids co-exist at any given time. The details of various biosolids with different plant locations, biosolids production processes, drying methods, number of years stockpiled before pyrolysis, and treatment and contamination grade are highlighted in Table 1 [4,17].

The commercial furnace employed in this study had fixed upper and lower temperature zones (i.e., 500 °C and 700 °C), which enabled the production of biochars at two different temperatures for use in this study.

A one-way ANOVA analysis was carried out to interpret the results from various biosolids and biochars prepared at 500 °C and 700 °C. The study determined the range and average properties of the biochars produced from various biosolids, with the hypothesis that they could be applied to land as a mix.

## 2. Materials and Methods

### 2.1. Biosolids

A total of eight biosolids samples were obtained from Pakenham, Somers, Boneo, and Mt. Martha wastewater treatment plants operated by South East Water (Victoria, Australia) during February 2016. The plants are located in the southeast of Melbourne, Victoria in Australia. These biosolids were sampled as they were representative of the range of biosolids currently produced at South East Wastewater treatment plants in Victoria. These plants predominantly receive domestic sewage and treat sewage sludge through an activated sludge process, aerobic/anaerobic digestion, and subsequent stockpiling of harvested biosolids from either outdoor drying pans or indoor drying sheds (see Table 1). The annual median inflow of wastewater to Somers, Pakenham, Boneo, and Mt. Martha WwTPs is 4, 6, 10, and 12 ML day^−1^, respectively.

Table 1 shows the eight biosolids (BS) sampled for this study. These biosolid samples were air-dried for 4 weeks and subsequently passed through a 2-mm sieve prior to producing biochars.

### 2.2. Biochars

Pyrolysis was conducted for all eight biosolids by means of the commercial ‘CharMaker’ (a mobile pyrolysis facility) operated by Earth Systems. Pyrolysis experiments were duplicated for each biosolids sample. Each biosolids sample was pyrolysed at two different temperatures, i.e., 500 °C and 700 °C. For each temperature, the results obtained from duplicate experiments were averaged and their standard deviations are reported using SPSS (Statistical Package for the Social Sciences) 15.0.

**Figure 1:**
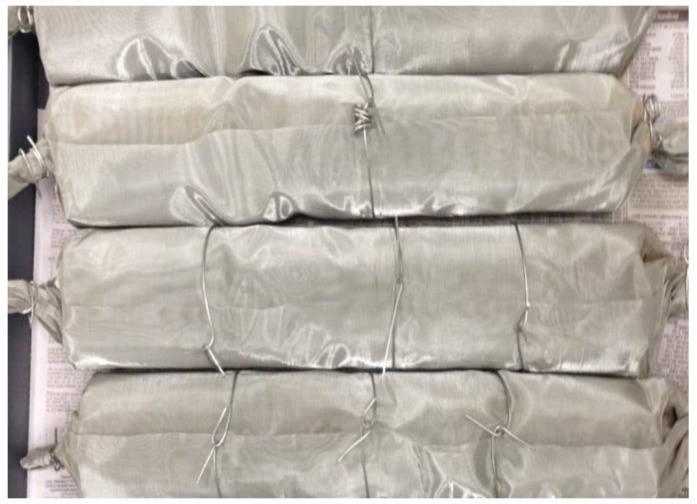
Stainless-steel mesh rolls with biosolids inside.

**Figure 2:**
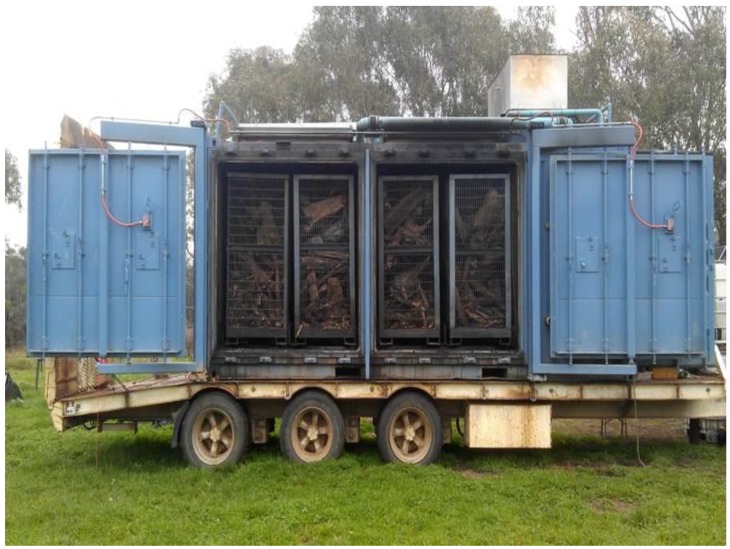
CharMaker commercial facility used for pyrolysing biosolids in this study.

During trials, approximately 2 kg of each biosolids sample was transferred into stainless-steel mesh rolls (Figure 1) with an aperture size of 0.1 mm and diameter of 8 cm and inserted into the commercial CharMaker (Figure 2) to allow the full penetration of heat into the roll centres during pyrolysis. All biochars were produced with the same residence time of 5 h and an external heating rate of 5 °C/min. In the remainder of the manuscript, biochars produced at 500 °C and 700 °C are referred to as biochars (BC) prepared at low temperature (BCL) and at high temperature (BCH).

### 2.3. Biosolids and Biochar Characterisation

#### 2.3.1. General Analyses

Electrical conductivity (EC) was measured using standard Method 3A1 [18]. In brief, 1:5 (*w*/*v*) sample/Milli-Q water suspensions were prepared and shaken for 1 h on a mechanical shaker and the EC was recorded on the clear supernatant solution after approximately 30 min. The pH of the supernatant solution was measured according to Method 4B2 [17].

Total C and N analysis was carried out using a combustion method in a C-230 LECO Analyser.

Cation exchange capacity (CEC) was determined using Method 15C1 [18]. Soluble salts were removed with 60% ethanol and 20% aqueous glycerol as a pre-treatment process if the sample EC was over 300 µS/cm. Cation extraction was carried out with 1M NH_4_Cl at a sample/solution ratio of 1:20. The exchangeable cations were determined by ICP-AES from the leachate, and the CEC was measured as NH^+^_4_ displaced with a 15% solution of KNO_3_ plus 6% Ca(NO_3_)_2_·4H_2_O.

Biochars’ and biosolids’ surface areas were determined using a gas sorption analyser, ASAP2000, using the nitrogen adsorption data. The sample was dried at 110 °C overnight for degassing. The equilibration interval for nitrogen adsorption was set at 30 s in the analyser, and the surface area was measured based on selected adsorption data using the Brunauer-Emmett-Teller BET method with Micromeritics software (Micrometrics SE Premium, v2.7).

#### 2.3.2. Total Elements

Total K, total P, total Ca, total Mg, and heavy metals in biosolids and biochars were analysed according to Method 17A1 [17]. Samples were oven-dried at 105 °C prior to analyses.

#### 2.3.3. Olsen P

Olsen P was determined by extraction with 100 mL of 0.5 M NaHCO_3_ at a pH of 8.5, using a 1:20 sample/solution ratio, and equilibrated for 30 min using a mechanical shaker. Extracts were filtered and analysed for Olsen P according to Method 9C2b [18].

#### 2.3.4. DTPA-Extractable Metals

The extractable metals were determined according to Method 12A1 [18]. Extraction with DTPA has been widely accepted to assess the readily exchangeable and more persistently bound metals, as this chelating agent has the ability to remove metal cations adsorbed by the mineral fraction and fixed in the organic and organometallic complexes [18]. One litre (1 L) of the DTPA extracting solution was prepared from 1.97 g DTPA, 1.47 g calcium chloride dihydrate, and 14.92 g triethanolamine, then dissolved with Milli Q water and adjusted to a pH of 7.3 with hydrochloric acid. The 1:2 sample/soil mixtures were equilibrated on a mechanical shaker for 2 h. The extracts were then filtered and digested with 2% nitric acid using a dilution factor (DF) of 100. The extractable metal concentrations of Cd and Pb were measured by ICP-MS, while Cu and Zn were determined using ICP-AES.

### 2.4. Thermogravimetric Analysis (TGA)

Ash content was determined using a Perkin Elmer TGA 4000 (Perkin-Elmer Inc., Wellesley, MA, USA). Samples were heated from 30 to 800 °C in an N_2_ atmosphere at a heating rate of 20 °C/min. The ash content was estimated as the final weight of the samples, when they were combusted under air at 800 °C.

### 2.5. Statistical Analysis

Statistical analyses were conducted with SPSS version 16.0. A one-way ANOVA analysis using the type of material (feed material biosolids, biochar prepared at 500 °C, and biochar prepared at 700 °C) was used to interpret the results. A post hoc Tukey’s HSD test was conducted after one-way analysis of variance. Homogeneity of variances was checked with Levene´s test. The assumption of normality was tested with the Kolmogorov-Smirnov test. When required, the studied parameters were log-transformed in order to achieve homogeneity of variances and a normal distribution of data. Data in Table 2, Table 3, Table 4 and Table 5 show non-transformed values. Mean values were deemed to be significantly different when *p* < 0.05.

## 3. Results and Discussion

Overall, a wide range in the properties of feedstock biosolids was observed as highlighted in Table 2. The trend also continued for both BCL and BCH. This was expected due to the disparity in biosolids as showcased in Table 1. The lower range for the properties might be related to clay biosolids (due to dilution), while the upper range might be attributed to pure biosolids. Studying this disparity was outside the scope of this study as the research was designed to investigate the on-site biosolids resources holistically to assess pyrolysis as a viable biosolids management option.

It is worth noting that the differences in feedstock biosolids are primarily attributed to the drying process used (see Table 1). In the sub-sections below, average values with standard deviations are discussed with an aim to also study the effect of pyrolysis temperature.

### 3.1. General Properties (pH, EC, C, N, CEC, Ash, and Surface Area)

The general properties of biosolids and corresponding biochars are tabulated in Table 3. The average pH of the biosolids used in this study was 5.84 ± 0.53. All biochars (i.e., BCL and BCH) produced were neutral to alkaline with an average pH of 7.68 ± 0.55 for BCL and 5.51 ± 0.49 for BCH, similar to the values reported in the literature (see Table 2), which established that even mild to strong acidic biosolids can be transformed into neutral to alkaline biochars at temperatures of 500–700 °C. No particular temperature effect was observed in the final pH of the biochars. These results are in contrast with other studies which found an increase in the pH of biochars with temperature as a consequence of the decrease of acidic surface groups during thermal treatment [25,26]. This may be attributed to the fact that the majority of the acidic surface groups might have already been released at 500 °C. Increased pH from pyrolysis could offer some degree of acid-neutralising capacity for acidic receiving soils when biochar is applied to agricultural land. This would generally result in higher pH values in soil following biochar addition. For example, Méndez et al. [12] reported an increase of soil pH by 0.2 units when different doses of biochar were applied on Mediterranean agricultural soils.

Average value of EC (a measure of salinity) in BS was 3.51 ± 0.68 dS/m, while average values of BCL and BCH were 0.82 ± 0.12 and 1.11 ± 0.15 dS/m, respectively (see Table 3). This may be related to the enriched inorganic ash contents with pyrolysis temperature [27]. The high EC values for biosolids could limit the range of crops suitable for cultivation in soils receiving these amendments. However, pyrolysis resulted in lower values of EC in the biochars, which widens the selection for crops with less tolerance to salinity [22].

The carbon content of the pyrolysed biosolids (i.e., biochars—BCL and BCH) were found to be lower compared to feedstock biosolids (see Table 3). The reduction in carbon may be attributed to the release of volatile carbon during pyrolysis. Similarly, the pyrolysis of biosolids led to a statistically significant reduction of the total N content in biochars. This can be attributed to the devolatilisation of nitrate, ammonia, and other volatile forms of N, processes which start at a temperature of around 200 °C [28]. The pyrolysis temperatures studied in this work were not found to result in major differences in the C and N contents in the biochars, as no clear trends were observed for low-temperature and high-temperature biochars. This could be attributed to the CharMaker operation or it is possible that low volatiles containing C and N might have all been released from the biosolids during pyrolysis at the lower temperature (i.e., 500 °C).

Cation exchange capacity (CEC) shows the ability of biochar to retain cations. Soils with high CEC values can retain cationic nutrient fertilisers (K^+^ and NH_4_^+^) in the root zone and thus prevent nutrient leaching. Biochars with a high CEC have the potential to improve soil productivity and reduce groundwater contamination via the retention of pollutants. In this work, CEC was statistically significantly lower in all biochars (i.e., BCL and BCH) compared to that in the feedstock biosolids. In particular, CEC was 28.9 ± 7.9 meq 100g^−1^ in the biosolids, 6.9 ± 1.1 meq 100g^−1^ in BCL, and 6.7 ± 1.2 meq 100g^−1^ in BCH. No clear effect of temperature was evident. The mechanisms governing the changes in CEC following pyrolysis are not well elucidated, with different articles reporting both decreases [27] and increases in CEC [6] after pyrolysis.

Biochars showed statistically significantly higher ash contents than their feedstocks, with average values increasing from 55.22% for BS to 65.22% and 74.60% for BCL and BCH, respectively.

The BET surface area was found to increase in the biochars when compared to the biosolids. The increase in biochar surface area is believed to be the result of the creation of pores and cracking in the biochars’ basal-structural sheets [28]. Gao et al. [29] also reported a correlation between the increase in biochar surface area and the pyrolysis temperature. This is due to the devolatilisation of organic and inorganic matters, responsible for opening the pores of the particles [26]. However, in this study, the surface areas of most biochars produced from biosolids remained approximately constant when the pyrolysis temperature was increased from 500 °C to 700 °C. This can be partly explained by the high proportion of ashes present in these types of biochars, as well as the mode of operation of the CharMaker, which could affect the filling and/or blocking of the biochar micro-pores [27].

### 3.2. Nutrients

Table 4 shows the results of total K, total P, Olsen P, total Ca, and total Mg for all biosolids and biochar samples.

The concentration of K in biochars (i.e., BCL, BCH) was found to be increased with respect to their corresponding biosolids. However, there was no temperature effects observed in the amounts of total K present in the final biochars. Generally, K is present in the form of minerals in biosolids and is retained along with biochars during pyrolysis, being non-volatile at the studied temperatures [27]. As a consequence, the average values of K were significantly higher in biochars (i.e., 3837 ± 1758 and 3687 ± 1691 mg/kg for BCL and BCH, respectively) when compared with biosolids (1617 ± 713 mg/kg), as other volatile matters were released during pyrolysis.

The average values of total P for biosolids and biochars were 11,975 ± 4521 mg/kg, 22,762 ± 20,364 mg/kg, and 24,494 ± 23,079 mg/kg for BS, BCL, and BCH, respectively. The relatively large amounts of total P in biochars may result from the high sorption capacity of biochar. P-release kinetics are largely dependent on P extractability [30].

Olsen P values were statistically significantly lower in BCL and BCH, with average values of 105 ± 44 and 115 ± 56 mg/kg, respectively, compared to 538 ± 386 mg/kg in BS. It is suggested that the Olsen P level in the biochars was intermediate and could act as a slow release fertiliser [31]. Lower Olsen P values in the biochars results in a soil amendment product more suitable for agricultural use than the parent biosolids. This is mainly due to variable nutrient content and imbalances in the N/P ratio in the feedstock biosolids. Moreover, as the biosolids guidelines [4] acknowledge that some soils may have a significant binding capacity for P, most land application schemes are based on meeting 100% crop N requirements (the replacement of N removed by the crop). This can potentially result in excess biosolids P applied to soils, resulting in increased soil P levels in excess of crop requirements and in P losses to surface waters (rivers, streams, and lakes), which can be a serious concern in some regions as elevated P concentrations can cause algae blooms and associated ecological impacts.

The total Mg in biochars was found to be statistically significantly increased (6244 ± 4056 mg/kg for BCL and 5969 ± 3874 mg/kg for BCH) with respect to their corresponding biosolids (3219 ± 1514 mg/kg). Again, there was no temperature effect observed on the amounts of total Mg present in the final biochars. No statistically significant differences were observed for total Ca between biosolids and biochars. Similar to K, nutrients such as Ca and Mg are also present in the form of minerals in biosolids and further retained along with biochars during pyrolysis, as they are non-volatile at the studied temperatures.

### 3.3. Heavy Metals

Table 5 shows the concentrations of all important heavy metals such as As, Cd, Cr, Cu, Pb, Hg, Ni, Se, and Zn in biosolids (BS) and biochars (BCL and BCH), as well as their allowable limits or range according to biosolids land contamination grade 1 (i.e., C1) and grade 2 (i.e., C2), and biochar guidelines for their application to receiving soil. Compared to other studies (Table 2), our biochars were in the mid to low range for As, Cr, Pb, Ni, and Se total contents. Heavy metals contents were found to be maintained at statistically similar levels in all biochars when compared to the corresponding feedstock biosolids, with the exception of Hg and Ni. Due to its lower boiling point, nearly all of the mercury was released during pyrolysis. The fate of Ni during the pyrolysis of biosolids needs further investigation, as it is affected by numerous variables including its total concentration, speciation, and the content of other elements in the biosolids [32]. Similar to the trend observed in other properties above, no effect of temperature was noted for the levels of heavy metals.

Despite statistically non-significant results for biochar metals in seven out of nine metals of importance, the absolute values of metals were considerably increased in most cases (Cu, Pb, and Zn in particular). This can be attributed to the loss of volatile matters from the feedstocks, which is expected. Overall, the levels (based on the absolute values for individual samples) of Cd, Cu, Se, and Zn would disqualify the biochars analysed in this study from being classified as grade C1. This is also true for biosolids, as higher initial values of all of the abovementioned heavy metals in addition to mercury in biosolids would disqualify these from being classified as grade C1. Grade C1 represents the lowest level of contaminants in Victoria and allows these products to be used without any specific management controls, while grade C2 requires controlled application. It should be noted that all biochars and biosolids investigated in this work still fall under grade C2. Another interesting observation was that, in spite of the biochars showing similar levels of heavy metals to the biosolids overall, the contents of heavy metals in the biochars were still found to be within the limits set up by the biochar guidelines of the International Biochar Initiative [33]. This suggests that biochar and biosolids management guidelines have major differences in terms of their permissible limits for land applications.

We believe that the total metal contents in biochars do not accurately represent the risk of plant uptake or mobility along the soil profile, and, as a consequence, the DTPA-extractable method was used to estimate the exchangeable and more persistently bound metals. The results of biosolids and biochar samples for extractable Cd, extractable Pb, extractable Cu, and extractable Zn are highlighted in Table 6. It was observed that, in general, pyrolysis resulted in a statistically significant decrease in the concentrations of DTPA-extractable Cd, Cu, Pb, and Zn, as compared to their corresponding feedstock biosolids (see Table 6). The pyrolysis temperature did not exert any specific effect on the DTPA-extractable levels of any heavy metals. The lower values of extractable heavy metals in biochar, compared to the parent biosolids, could be explained by the increase in biochar pH and surface areas that restrict the release of these metals [34,35,36]. Lu et al. [36] also reported that decreases in DTPA-extractable metals were due to the increases of P contents via pyrolysis to form phosphate precipitation with these metals. Another explanation is that the development of functional groups on the biochar surface immobilizes metals by forming organo-metallic complexes [37]. Functional groups in biochars, such as carbonyls, phenolic, hydroxyls carboxylates, and aromatic C=C, show variability with pyrolysis temperatures, type of process, and the nature of the feedstock [38]. Also, the combination of competition between the binding sites and the theoretical electrostatic repulsion between metal cations leads to differences in metal extractability after pyrolysis. Among these functional groups, aromatic C=C and carbonyls preferably bind Pb [39].

Biochar, as a soil amendment, has been proved to effectively reduce high concentrations of soluble metals such as Cd and Zn from contaminated soils [40]. Waqas et al. [11] reported that the addition of sewage sludge biochars leads to a significant decrease in in DTPA-extractable Pb, Cu, and Zn when compared to control soils when biochars were mixed with soils at biochar concentrations of 5% and 10%.

### 3.4. Significant Observations and Implications Derived from this Study

The biosolids from different wastewater treatment plants and with different management scenarios (Table 1) had varied physicochemical characteristics (Table 2). The total Cd, Cu, Se, and Zn in the biochars consistently exceeded the upper contaminant limits for receiving soils issued by EPA Victoria for the unrestricted use of biosolids (grade C1). However, it is important to note that despite the elevated total metal levels, the mobility, leaching risk, and bio-availability of heavy metals (measured as extractable metals in the current work) would be greatly reduced in biochars following pyrolysis compared to biosolids (Table 6). In fact, the biochars produced in this study had values of total metals within the limits proposed by the biochar initiative guidelines [33]. Ultimately, plant uptake of heavy metals will depend not only on biochar properties but also on the properties (pH, organic matter, CEC, texture) of the receiving soil. Thus, an immediate implication of our study is that more research, both at the laboratory and at the field levels, will be required in order to evaluate safe levels of applications for these types of biochars in soils with different characteristics. Olsen P was also reduced significantly in the biochars (Table 4), leading to a lower risk of P run-off from the land application of these materials. From the current work, it is clear that there is scope for altering the regulations concerning the land application of biochar produced from biosolids and that these regulations should be based on extractable or leaching metals rather than on total contents. However, it should be noted that comprehensive experimental work needs to be conducted before proposing any changes to regulations. The current work should only serve as a reference in recommending further study to evaluate the feasibility of altering the regulations around the use of biochars produced from biosolids for land application. Moreover, it still remains unknown whether the degradation or other factors in the soil environment of biochars produced from biosolids could increase the long-term release of detrimental or harmful elements. The long-term fate of pollutants and nutrients in biochar should be established in different soil types and with biochars prepared at different pyrolysis temperatures.

## 4. Conclusions

The current work was focused on converting biosolids into biochar to assess changes in physicochemical properties. It is concluded that pyrolysis has the potential to deliver promising benefits in biosolids management for wastewater industries. From an economic perspective, pyrolysis (following the dewatering of sewage sludge) can reduce the significant costs associated with biosolids stockpiling and storage. The physicochemical properties (e.g., pH, EC, Olsen P, and surface area) of biochars derived from biosolids are more amenable for land application programs when compared to that of feedstock biosolids. Pyrolysis was also able to significantly reduce the extractable contaminant concentration for Cd, Cu, Pb, Zn, and P, which indicated that the land application of biochars would pose lower risks than the application of biosolids. Overall, this study highlighted the potential for a pyrolysis step to be incorporated into the biosolids disposal process at wastewater treatment facilities, which could greatly reduce or eliminate the environmental concerns associated with current land application strategies for biosolids in southeast Victoria and worldwide.

## Figures and Tables

**Table 1:** Wastewater treatment plant (WwTP) location, biosolids production details, and biosolids grades.

WwTP Location	Biosolids Production Process	Number of Years Stockpiled (Stored) ^1^ before Pyrolysis	Treatment (T) Grade and Contamination (C) Grade	Biosolids Drying Process
Pakenham	Waste activated sludge from primary/secondary treatment-sludge lagoon-sludge drying pan (clay biosolids storage) ^1^	3	T1C2	Outdoor air drying in clay-lined sludge drying pans. Biosolids inadvertently mixed with clay during the mechanical sludge drying process in the drying pans.
		3	T1C2	
Somers	Waste activated sludge from sequential batch reactors-sludge lagoon-sludge drying pan (clay biosolids storage) ^1^	3	T1C2	
Boneo	Waste activated sludge from bioreactor-aerobic digester-sludge lagoon-sludge drying pan (clay biosolids storage) ^1^	2	T1C2	
		3	T1C2	
	Waste activated sludge from bioreactor-aerobic digester-sludge lagoon-belt press-solar dryer shed (pure biosolids storage) ^1^	1	T1C2	Indoor air drying in concrete-lined sludge drying sheds. Biosolids without clay contamination.
	Waste activated sludge from bioreactor-aerobic digester-sludge lagoon-belt press-solar dryer shed (pure biosolids with no storage)	0	T3C2	
Mt Martha	Waste activated sludge from anaerobic digester-centrifuge-solar dryer shed (pure biosolids with no storage)	0	T3C2	

^1^ The biosolids produced after 1–3 years of storage are of high quality (T1 grade) and appropriate for a range of beneficial reuse options. One year of stockpiling is not a prescribed treatment process to produce T1 biosolids according to EPA Victoria’s biosolids guidelines [4]. However, South East Water obtained approval from EPA Victoria in December 2015 to use 1- and 2-year-old biosolids stockpiles from Boneo and Somers WwTPs as T1 grade biosolids. This approval was based on a research study that showed that sludge treatment and management processes operating at Boneo and Somers WwTPs exceeded the verification requirements for alternative treatment processes to produce T1 grade biosolids with respect to prescribed faecal bacterial numbers and enteric virus reduction after stockpile storage for a minimum of 1 year [17].

**Table 2:** Variability in the physicochemical properties of the biosolids (BS) and biochars prepared at low (BCL) and high (BCH) temperatures. The three middle columns show the data in our study, while the data in the last column were obtained from References [11,12,13,16,19,20,21,22,23,24]. Values for some properties were not provided in more than an article and are reported in the table as NA.

Property	Range of Variation for BS	Range of Variation for BCL	Range of Variation for BCH	Biosolids Biochar in Other Studies
pH	3.9–6.7	6.1–9.1	6.2–8.6	5.8–9.54
EC (dS/m)	1.4–5.8	0.5–1.3	0.8–1.8	0.5–5.4
C (%)	4.7–33.0	1.8–30.3	2.4–30.9	14–77
N (%)	0.6–5.1	0.13–2.49	0.18–3.05	0.3–7.1
CEC (meq/100 g)	11.0–53.0	2.9–11.0	4.1–11.0	2.3–12
Ash (%)	45–65	55–75	58–73	26–79
Surface area (m^2^/g)	3.9–29.7	25.4–136.0	28.3–143.0	4–90
Total K (mg/kg)	1000–3200	2100–8200	1700–7500	800–6470
Total P (mg/kg)	3700–25,000	4300–59,000	4000–71,000	66,000–124,000
Olsen P (mg/kg)	180–1300	32–190	38–230	NA
Total Ca (mg/kg)	3300–35,000	3800–92,000	3400–110,000	37,000–90,000
Total Mg (mg/kg)	2000–5500	2500–13,000	2300–12,000	13,000–50,000
Total As (mg/kg)	5.0–9.0	5.0–9.0	5.0–9.0	5.2–16.7
Total Cd (mg/kg)	0.4–1.8	0.5–3.1	0.5–3.4	0.27–8.8
Total Cr (mg/kg)	16–53	38–58	36–65	25–281
Total Cu (mg/kg)	110–777	95–1500	100–1300	222–1000
Total Pb (mg/kg)	18–48	25–70	25–72	54–168
Total Hg (mg/kg)	0.3–1.1	0.05–0.05	0.05–0.05	0.01–0.40
Total Ni (mg/kg)	12–25	20–360	28–120	0–635
Total Se (mg/kg)	3–5	3–5	3–6	9.7–14
Total Zn (mg/kg)	200–1100	210–2300	210–2300	250–2940
DTPA-Cd (mg/kg)	0.1–1.0	0.01–0.17	0.01–0.28	NA
DTPA-Cu (mg/kg)	14–475	25–96	23–70	NA
DTPA-Pb (mg/kg)	0.1–4.3	0.2–2.0	0.2–0.9	NA
DTPA-Zn (mg/kg)	52–555	7–52	9–89	NA

**Table 3:** General properties of the biosolids and corresponding biochar samples. BS: biosolids, BCL: biochars prepared at low temperature, BCH: biochars prepared at high temperature. Different letters within the same row represent significant differences in the mean values.

General Properties	BS	BCL	BCH
pH	5.84 ± 0.53 a	7.68 ± 0.55 b	7.51 ± 0.49 b
EC (dS/m)	3.51 ± 0.68 a	0.82 ± 0.12 b	1.11 ± 0.15 c
C (%)	14.6 ± 5.8 a	10.4 ± 6.1 b	11.2 ± 6.1 b
N (%)	2.22 ± 0.96 a	0.85 ± 0.53 b	1.04 ± 0.62 b
CEC (meq/100 g)	28.9 ± 7.9 a	6.9 ± 1.1 b	6.7 ± 1.2 b
Ash (%)	55.22 ± 6.08 a	65.22 ± 7.09 b	74.60 ± 7.97 c
Surface area (m^2^/g)	15.8 ± 7.4 a	53.7 ± 30.4 b	54.2 ± 29.1 b

**Table 4:** Nutrient contents (mg/kg) of the biosolids and corresponding biochar samples. BS: biosolids, BCL: biochars prepared at low temperature, BCH: biochars prepared at high temperature. Different letters within the same row represent significant differences in the mean values.

Nutrients	BS	BCL	BCH
Total K	1617 ± 713 a	3837 ± 1758 b	3687 ± 1691 b
Total P	11,975 ± 4521 a	22,762 ± 20,364 b	24,494 ± 23,079 b
Olsen P	538 ± 386 a	105 ± 44 b	115 ± 56 b
Total Ca	12,620 ± 9579 a	14,950 ± 10,340 a	15,230 ± 10,935 a
Total Mg	3219 ± 1514 a	6244 ± 4056 b	5969 ± 3874 b

**Table 5:** Total metal concentrations (mg/kg) of the biosolids and corresponding biochar samples. BS: biosolids, BCL: biochars prepared at low temperature, BCH: biochars prepared at high temperature. Different letters within the same row represent significant differences in the mean values.

Metals	BS	BCL	BCH	C1 Grade ^1^	C2 Grade ^1^	Biochar Guidelines ^2^
As	6.5 ± 1.7 a	6.9 ± 1.8 a	6.6 ± 1.6 a	20	60	13–100
Cd	1.18 ± 0.57 a	1.54 ± 0.89 a	1.71 ± 1.03 a	1	10	1.4–39
Cr	36 ± 11 a	46 ± 6 a	45 ± 7 a	400	3,000	93–1200
Cu	313 ± 225 a	464 ± 445 a	431 ± 411 a	100	2,000	143–6000
Pb	27 ± 9 a	43 ± 16 a	45 ± 7 a	300	500	121–300
Hg	0.79 ± 0.32 a	0.05 ± 0.00 b	0.13 ± 0.17 b	1	5	1–17
Ni	19 ± 4 a	86 ± 94 b	58 ± 39 b	60	270	47–420
Se	3.6 ± 0.9 a	3.5 ± 0.7 a	4.0 ± 1.0 a	3	50	2–200
Zn	552 ± 360 a	913 ± 817 a	888 ± 803 a	200	2500	416–7400

^1^ EPA Victoria Biosolids Guidelines [4], ^2^ International Biochar Initiative Guidelines [33].

**Table 6:** DTPA-extractable concentrations (mg/kg) of Cd, Cu, Pb, and Zn in the biosolids and corresponding biochar samples. BS: biosolids, BCL: biochars prepared at low temperature, BCH: biochars prepared at high temperature. Different letters within the same row represent significant differences in the mean values.

DTPA-Extractable Metals	BS	BCL	BCH
Cd	0.491 ± 0.153 a	0.056 ± 0.028 b	0.080 ± 0.048 b
Cu	128 ± 76 a	45 ± 12 b	42 ± 9 b
Pb	1.44 ± 0.63 a	0.72 ± 0.28 b	0.51 ± 0.14 b
Zn	231 ± 81 a	26 ± 9 b	37 ± 15 b
